# Maturity Group Classification and Maturity Locus Genotyping of Early-Maturing Soybean Varieties from High-Latitude Cold Regions

**DOI:** 10.1371/journal.pone.0094139

**Published:** 2014-04-16

**Authors:** Hongchang Jia, Bingjun Jiang, Cunxiang Wu, Wencheng Lu, Wensheng Hou, Shi Sun, Hongrui Yan, Tianfu Han

**Affiliations:** 1 Ministry of Agriculture (MOA) Key Laboratory of Soybean Biology (Beijing), Institute of Crop Sciences, The Chinese Academy of Agricultural Sciences, Beijing, China; 2 Heihe Branch of Heilongjiang Academy of Agricultural Sciences, Heihe, Heilongjiang, China; Nanjing Forestry University, China

## Abstract

**Background:**

With the migration of human beings, advances of agricultural sciences, evolution of planting patterns and global warming, soybeans have expanded to both tropical and high-latitude cold regions (HCRs). Unlike other regions, HCRs have much more significant and diverse photoperiods and temperature conditions over seasons or across latitudes, and HCR soybeans released there show rich diversity in maturity traits. However, HCR soybeans have not been as well classified into maturity groups (MGs) as other places. Therefore, it is necessary to identify MGs in HCRs and to genotype the maturity loci.

**Methods:**

Local varieties were collected from the northern part of Northeast China and the far-eastern region of Russia. Maturity group reference (MGR) soybeans of MGs MG000, MG00, and MG0 were used as references during field experiments. Both local varieties and MGR soybeans were planted for two years (2010-2011) in Heihe (N 50°15′, E 127°27′, H 168.5 m), China. The days to VE (emergence), R1 (beginning bloom) and R7 (beginning maturity) were recorded and statistically analyzed. Furthermore, some varieties were further genotyped at four molecularly-identified maturity loci *E1*, *E2*, *E3* and *E4*.

**Results:**

The HCR varieties were classified into MG0 or even more early-maturing. In Heihe, some varieties matured much earlier than MG000, which is the most early-maturing known MG, and clustered into a separate group. We designated the group as MG0000, following the convention of MGs. HCR soybeans had relatively stable days to beginning bloom from emergence. The HCR varieties diversified into genotypes of *E1*, *E2*, *E3* and *E4*. These loci had different effects on maturity.

**Conclusion:**

HCRs diversify early-maturing MGs of soybean. MG0000, a new MG that matures much earlier than known MGs, was developed. HCR soybean breeding should focus more on shortening post-flowering reproductive growth. *E1*, *E2*, *E3*, and *E4* function differentially.

## Introduction

Soybean, a short-day crop of significant economic and ecological importance, diversifies significantly during maturity. Maturity traits not only include total growth duration but also the vegetative and reproductive phases, and their relative ratios [1], [2], reflecting whether temperature and light conditions satisfy the growth of soybean [3] and determine the adaptation of soybean varieties.

Maturity is controlled by multiple loci or genes [4]. Nine maturity loci (*E1*–*E8*, and *J*) have been reported [5]–[13], have different effects on soybean flowering and play different roles in photo-thermal responses [9], [14], [15]. Four of these loci have been molecularly identified with map-based or candidate-based cloning. *E1* encodes a soybean-specific potential transcription factor [16], *E2* is a *GIGANTEA* homologue of *GmGIa* [17], and *E3* and *E4* are the *GmPhyA3* [18] and *GmPhyA2* [19] phytochromes, respectively. More of these have been reviewed by Xia et al. [20]. Although some flowering genes are not included in or molecularly identified as maturity loci, they might also function in maturity. For example, *GmFT2a* displays differential transcriptional profiles under different temperatures and photoperiod conditions in two varieties with totally different photoperiod sensitivities [21]; however, its polymorphism appears to not be related to the maturity diversification [22]. Therefore, the underlying mechanism of maturity diversity is not yet clear.

Categorizing soybeans into different “maturity groups (MGs)” is convenient for breeding practices [23]. In North America, a 13-maturity group classification system has been set up according to the latitudes of adaptation [24]–[26]. In contrast, Chinese soybean researchers have divided varieties into twelve MGs based on the environments and planting patterns in China [27]–[29]. However, this classification method is not unified. Gai et al. classified Chinese soybeans into twelve maturity groups based on a maturity group distance of 10–15 days [29]. Wu et al. used the average of the neighboring maturity groups as the threshold to classify maturity groups [30], whereas Alliprandini et al. used a linear regression method to categorize local soybeans [31]. These different methods support the idea that the maturity mechanism is rather complex in soybeans and that diversified environments have significant impacts. However, in high-latitude cold regions (HCRs), little is known.

Since the development of soybean breeding and plantations, HCRs have become important for soybean production. Soybean production HCRs are predominantly located in China, Russia and North America. In China, they are located in the Heihe, Yichun, Daxing'anling region, North Qiqiha'er and North Suihua of Heilongjiang Province and Hulunbei'er of Inner Mongolia, which are predominantly located between N47°–53°34′. In these regions, soybean acreage reaches over 1,000,000 ha [32]. In Russia, the far-eastern region, especially the Amurskaya Oblast, which neighbors the Heilongjiang province of China, is the main region for soybean production [33], accounting for 90% of Russian soybean production [34]. In Canada, soybean production has expanded from southern Ontario in the 1970s to eastern Ontario, Quebec and Manitoba, reaching 1.7 million ha in 2012 (http://www.soybeancouncil.ca/). HCRs will become more important for soybean production, especially under the background of climate change.

In HCRs, the environment changes more violently across latitudes and between seasons than at lower latitudes, especially for temperature and photoperiod. In these areas, soybean has a short growth duration and is relatively insensitive to photo-thermal regimes [35]. Here, we use MGR soybeans, MG0, MG00 and MG000, as a reference to classify local HCR soybeans into different maturity groups and to genotype them at four molecularly identified maturity loci, *E1*, *E2*, *E3* and *E4*, to learn the adaptation mechanism of soybean in extreme environments.

## Materials and Methods

### 1 Plant materials

Ninety-three soybean varieties were involved in this study (Table 1). Of these varieties, 9 were MGR soybeans (MG000, MG00, and MG0) from North America; 6 were MGR varieties (MG000, MG00 and MG0) from China; 18 were from Amurskaya Oblast (Russia); and the remaining 60 were released varieties of HCRs from China.

**Table 1:** Soybean varieties and their maturity groups (MGs)

	MGR (maturity group reference) varieties		Varieties from high-latitude cold regions with proposed MG	
Maturity group	North America	China	High-latitude cold region in Northeast China	Amurskaya Oblast in Russia
**MG0000** *			Dongnong 36, Dongnong 41, Dongnong 41-C†, Hujiao 07-2123, Hujiao 07-2479†, Lingbei 8†	Paula†, R-3†, R-4, Star 4/75†, Sunset†
**MG000**	Maple Presto, OAC Vision	Heihe 12	Dengke 2, Ha 6223-4, Heihe 11, Heihe 14, Heihe 20, Heihe 28, Heihe 35, Heihe 41, Heihe 44, Heihe 49, Kennong 8	Bista, R-2, Sonata
**MG00**	Canatto, Maple Ridge, Glacier	Heihe 3, Heihe 8	Bei 02-7495, Beidou 16, Beidou 24, Beifeng 1, Dongnong 40, Fengshou 23, Hefeng 37, Heihe 5, Heihe 7, Heihe 9, Heihe 13, Heihe 33, Heihe 37, Heihe 39, Heihe 45, Heihe 50, Heihe 51, Huajiang 2, Jiufeng 7, Jiufeng 10, Mengdou 7, Mengdou 9, Mengdou 11, Mengdou 31	Amur 262, Amur 283, Dewdrop, Gritiaz 80, Harmony, Lydia, October Revolution 70, R-1, Sunset 1, Terek
**MG0**	MN0201, MN0901, Surge, Traill	Jilin 30, Jiunong 21, Suinong 14	Bei 1249, Beidou 8, Beidou 19, Beidou 37, Fengshou 15, Fengshou 24, Fengshou 26, Fengshou 27, Heihe 18, Heihe 36, Heihe 38, Heihe 43, Heihe 46, Heihe 48, Huajiang 3, Jiangmodou 1, Jiufeng 9, Mengdou 30, Zhongzuo GHJ90962	

*MG0000 is newly proposed here.

† Proposed MG reference soybeans for MG0000.

### 2 Field experiments

Field experiments were conducted between 2010 and 2011 in the experimental field of Heihe Branch of Heilongjiang Academy of Agricultural Sciences (N 50°15′, E 127°27′, H 168.5 m). No specific permissions were required for these locations/activities. No endangered or protected species were involved. On May 9^th^ of each year of these two years, the soybeans were manually sowed in two rows (row length of 2 m, plant spacing of 5 cm, and row spacing of 60 cm). After emergence, they were thinned for 30 uniform healthy plants. The days reaching VE (emergence), R1 (beginning bloom) and R7 (beginning maturity) were recorded in line with Fehr and Caviness [36].

### 3 Genotyping of *E1*, *E2*, *E3* and *E4*

Fresh leaves were used to isolate DNA using the TIANGEN (Beijing, China) New Plant Genomic DNA Isolation kit (DP320-03). Loci *E1*, *E2*, *E3* and *E4* were genotyped following previously described methods [16]–[19].

### 4 Statistical analysis

In R environment [37], APCluster [38] was used to cluster varieties into different maturity groups with negative Euclidean distances as mutual pairwise similarities and with the maturity duration data in 2010 and 2011 as input. A principal component analysis (PCA) was also conducted in FactoMineR [39] with default settings.

## Results

### Tested soybean varieties matured differently with different beginning bloom days

**Figure 1:**
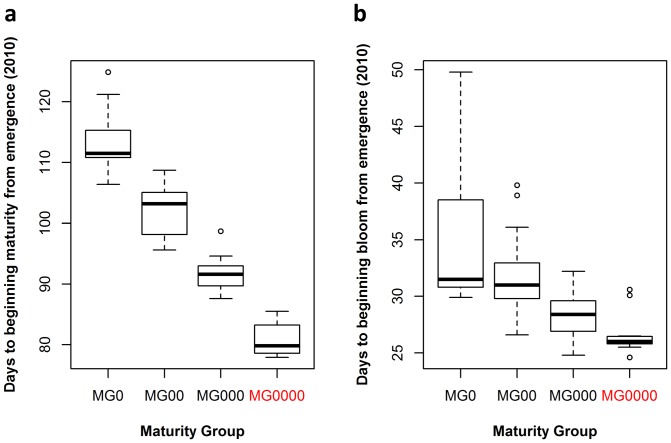
Days to beginning maturity and beginning bloom from emergence in 2010. **(a).** Days to beginning maturity from emergence (2010). **(b).** Days to beginning bloom from emergence (2010). MG0000, a new proposed maturity group, is marked in red.

A high latitude with a long photoperiod and low average temperature significantly affected the maturations rates of the soybeans (shown in Table S1 and File S1). For the MGR soybeans (MG000 – MG0), MG0 diverged with some varieties maturing and some not, while MG00 and MG000 matured (Table 2). The matured MGR soybeans matured 82.0 to 124.9 days after emergence (Table 2), and their MGs significantly differed at the day to maturity after emergence (Figure 1a). Moreover, the days to beginning bloom from emergence (DFF) also diversified significantly (Figure 1b). MG was the more early-maturing with the range of DFF narrowed even more (Figure 1b).

**Table 2:** Days to beginning maturity from emergence for the MGR (maturity group reference) soybeans in Heihe, China.

		Days to beginning maturity from emergence	
Variety	MG	2010	2011
**Maple Presto (E)**	**MG000**	88.5±0.9	82.0±1.4
**OAC Vision (L)**		93.8±0.4	87.8±0.9
**Heihe 12**		94.6±0.9	85.9±1.5
**Canatto (E)**	**MG00**	97.8±0.9	92.0±0.8
**Maple Ridge (E)**		95.9±0.8	96.3±0.6
**Glacier (L)**		107.3±1.1	95.2±0.8
**Heihe 3**		102.4±1.2	97.8±0.9
**Heihe 8**		97.3±1.1	90.8±0.6
**MN0201 (E)**	**MG0**	120.4±1.0	99.4±0.7
**Traill (E)**		113.1±1.2	99.1±0.7
**MN0901 (L)**		124.9±1.2	115.4±0.6
**Surge (L)**		R6	R5
**Suinong 14**		R6	R5
**Jilin 30**		R4	
**Jiunong 21**			

Soybeans with brackets are maturity group reference soybeans from North America. The others are from China. Letters E and L (in brackets) indicate that the soybean is relatively early- or late-maturing, respectively, in the corresponding maturity group; R4, R5 and R6, the growth stages of soybeans failing to mature at first frost.

For the other non-MGR soybeans, they also exhibited significant variations in their maturity, with a range of 43–54 days (Table S1). Huajiang 3 (2010), and Beidou 37 and Zhongzuo GHJ90962 (2011) matured last in different years, whereas Sunset (2010), and R-4 (2011) were the earliest to mature (Table S1). However, 11 varieties matured even ealier than MG000 during the two years, that is, R-4, Star 4/75, Hujiao 07-2123, Sunset, Hujiao 07-2479, R-3, Dongnong 36, Paula, Dongnong 41, Lingbei 8, and Dongnong 41-C (Table 3). Dengke 2 matured earlier than the MGR soybeans MG000 in 2010 but later than the early MG000 reference varieties in 2011 (Table S1).

**Table 3:** Soybean varieties earlier than the MGR (maturity group reference) soybeans of MG000 in terms of days to beginning maturity from emergence.

Variety	2010	2011	Variation/Mean
**R-4**	78.3±0.5	72.1±0.9	0.08
**Star 4/75** *	78.2±0.5	73.3±1.3	0.06
**Hujiao 07-2123**	79.1±1.2	74.0±1.4	0.07
**Sunset** *	77.9±0.8	74.2±0.9	0.05
**Hujiao 07-2479** *	78.9±1.0	76.3±0.5	0.03
**R-3** *	80.0±0.5	76.6±0.9	0.04
**Dongnong 36**	85.5±0.9	77.3±0.7	0.10
**Paula** *	79.8±0.7	77.8±1.6	0.03
**Dongnong 41**	84.3±1.1	77.9±0.6	0.08
**Lingbei 8** *	82.5±0.7	79.1±0.7	0.04
**Dongnong 41-C** *	84.0±0.8	81.8±0.7	0.03
**MGR soybeans, MG000**	88.5–94.5	81.9–87.8	NA

*MGR soybeans for proposed MG0000. NA, “not available”.

### The tested soybean varieties clustered into four maturity groups

**Figure 2:**
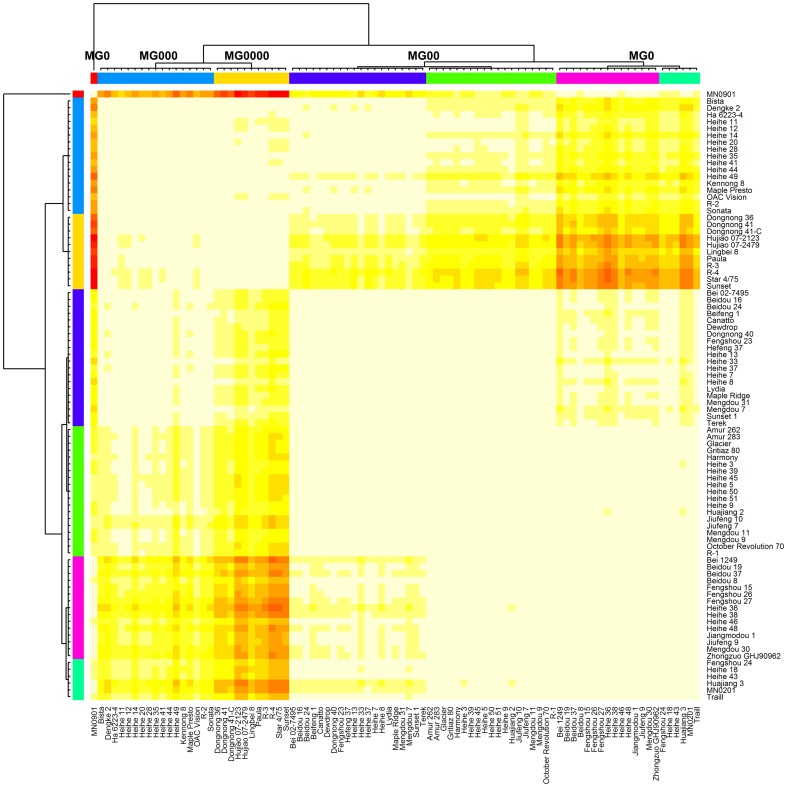
Heatmap for the clustering of soybeans from high-latitude cold regions.

**Figure 3:**
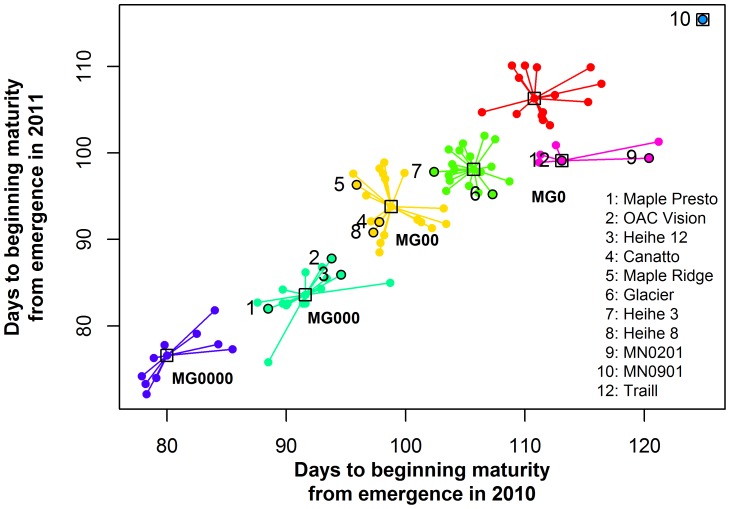
Clusters of soybeans from high-latitude cold regions. Numbers from 1 to 10 and 12 indicate different maturity group reference soybeans.

The tested soybean varieties consistently matured between years (Table 4). R1–R7 and VE-R7 displayed a high correlation in the same year, whereas VE-R1 did not correlate with the former two (Table 4). With APCluster [38], these soybeans were clustered into seven clades (Figures 2 and 3). For the MGR soybeans, MG0 and MG00 each clustered into two clades, whereas MG000 clustered into one clade (Figures 2 and 3). The varieties that matured earlier than MG000 during the two years all formed one clade (Figures 2 and 3). This clade is significantly different with the other MGs and has been designated as a new MG of MG0000 following the convention of MG. The other clade, without MGR soybeans, is located between the two MG0 clades; thus, it is also included in MG0. Thus, the tested varieties were clustered into four MGs (MG0, MG00, MG000, and MG0000, see Table 1). Based on the Variation/Mean (VE-R7) and the VE-R7 during the two years, MGR soybeans for MG0000 were proposed. They are Star 4/75, Sunset, Hujiao 07-2479, R-3, Paula, Lingbei 8, and Dongnong 41-C (Table 1).

**Table 4:** Correlation (R^2^) between maturity traits in different years.

2010	VE-R7	1					
	VE-R1	0.400	1				
	R1-R7	0.772	0.075^a^	1			
2011	VE-R7	0.854			1		
	VE-R1		0.658		0.321	1	
	R1-R7			0.702	0.891	0.108^a^	1
		VE-R7	VE-R1	R1-R7	VE-R7	VE-R1	R1-R7
		2010			2011		

(p<0.001) ^a^ p<0.01.

### Genotyping of the *E1*, *E2*, *E3* and *E4* maturity loci

Forty-eight varieties were genotyped for the maturity loci *E1*, *E2*, *E3* and *E4* (Table 5). Of those varieties, 7, 8, 21, and 12 were from MG0000, MG000, MG00, and MG0, respectively. Seven genotypes were identified, that is, e1e2e3e4 (4 varieties in 3 MGs), e1e2e3E4 (23 in 3), e1e2E3e4 (4 in 2), e1e2E3E4 (12 in 3), e1E2E3E4 (1 in 1), E1e2e3e4 (2 in 1), and E1e2e3E4 (2 in 1). Each MG diversified into *E1*, *E2*, *E3* and *E4* genotypes.

**Table 5:** Genotype of soybean varieties at the *E1*, *E2*, *E3* and *E4* loci.

Maturity Group	Genotype	Number	Variety
**MG0000**	e1e2e3e4	2	Hujiao 07-2123, Hujiao 07-2479
	e1e2E3e4	2	Dongnong 36, Paula*
	e1e2E3E4	2	R-4*, Star 4/75*
	e1E2E3E4	1	Sunset*
**MG000**	e1e2e3e4	1	Dengke 2
	e1e2e3E4	1	Heihe 12
	e1e2E3e4	2	Bista*, R-2*
	e1e2E3E4	4	Heihe 14, Heihe 35, Heihe 49, Sonata*
**MG00**	e1e2e3e4	1	Mengdou 31
	e1e2e3E4	12	Amur 262*, Dongnong 40, Fengshou 23, Hefeng 37, Heihe 3, Heihe 8, Heihe 9, Heihe 33, Heihe 45, Huajiang 2, Mengdou 11, R-1*
	e1e2E3E4	6	Amur 283*, Dewdrop*, Heihe 13, Lydia*, October Revolution 70*, Terek*
	E1e2e3e4	2	Harmony*, Gritiaz 80*
**MG0**	e1e2e3E4	10	Beidou 37, Fengshou 15, Fengshou 26, Fengshou 27, Heihe 18, Heihe 38, Heihe 43, Huajiang 3, Mengdou 30, Sunset 1*
	E1e2e3E4	2	Jiangmodou 1, Heihe 36

*Russian varieties.

### *E1*, *E2*, *E3* and *E4* maturity loci are diverse in maturity groups

**Figure 4:**
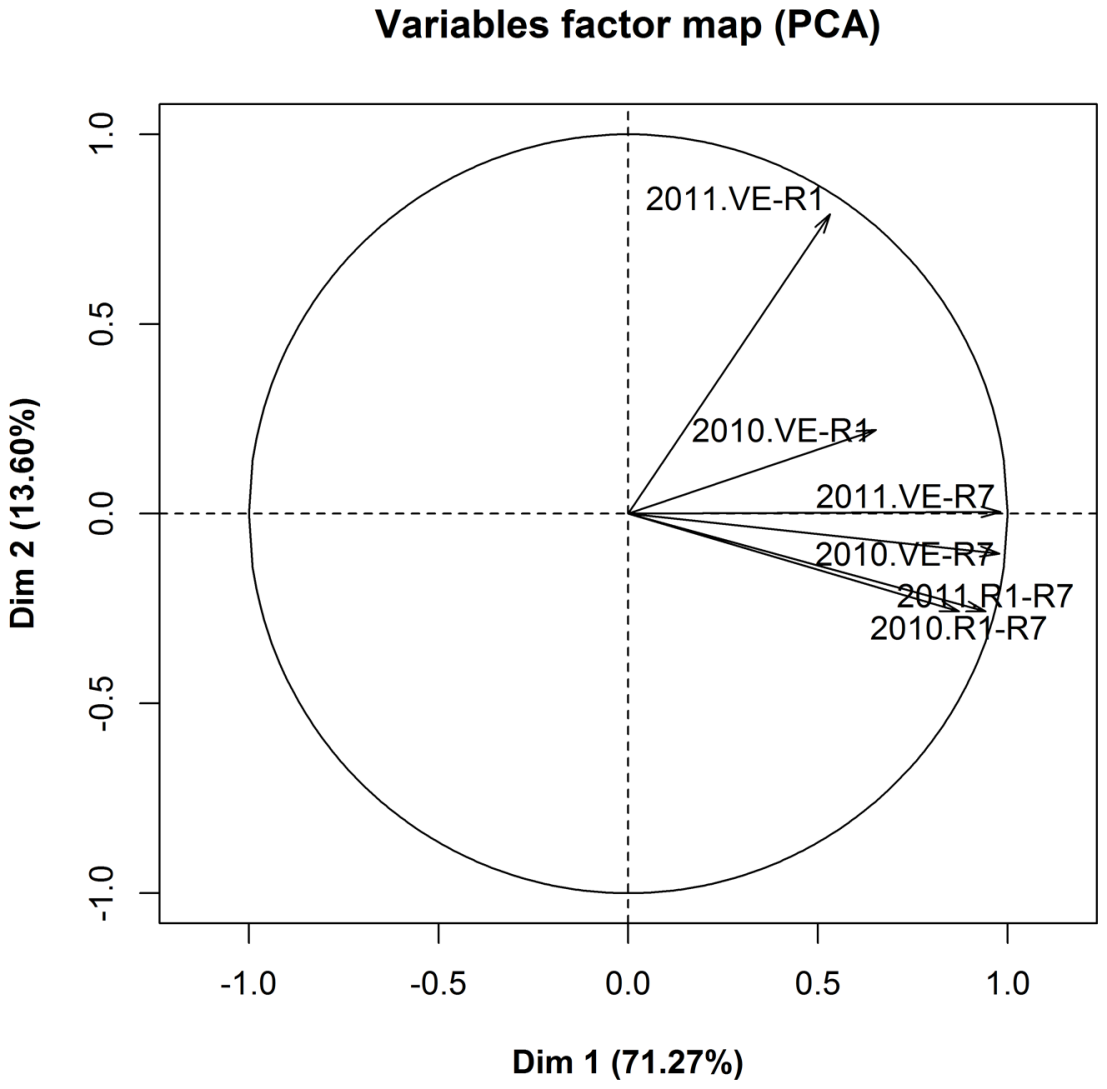
PCA variable factor map of soybeans from the high-latitude cold regions. 2010. VE-R7 is the days between VE and R7 in 2010. Others are similar.

**Figure 5:**
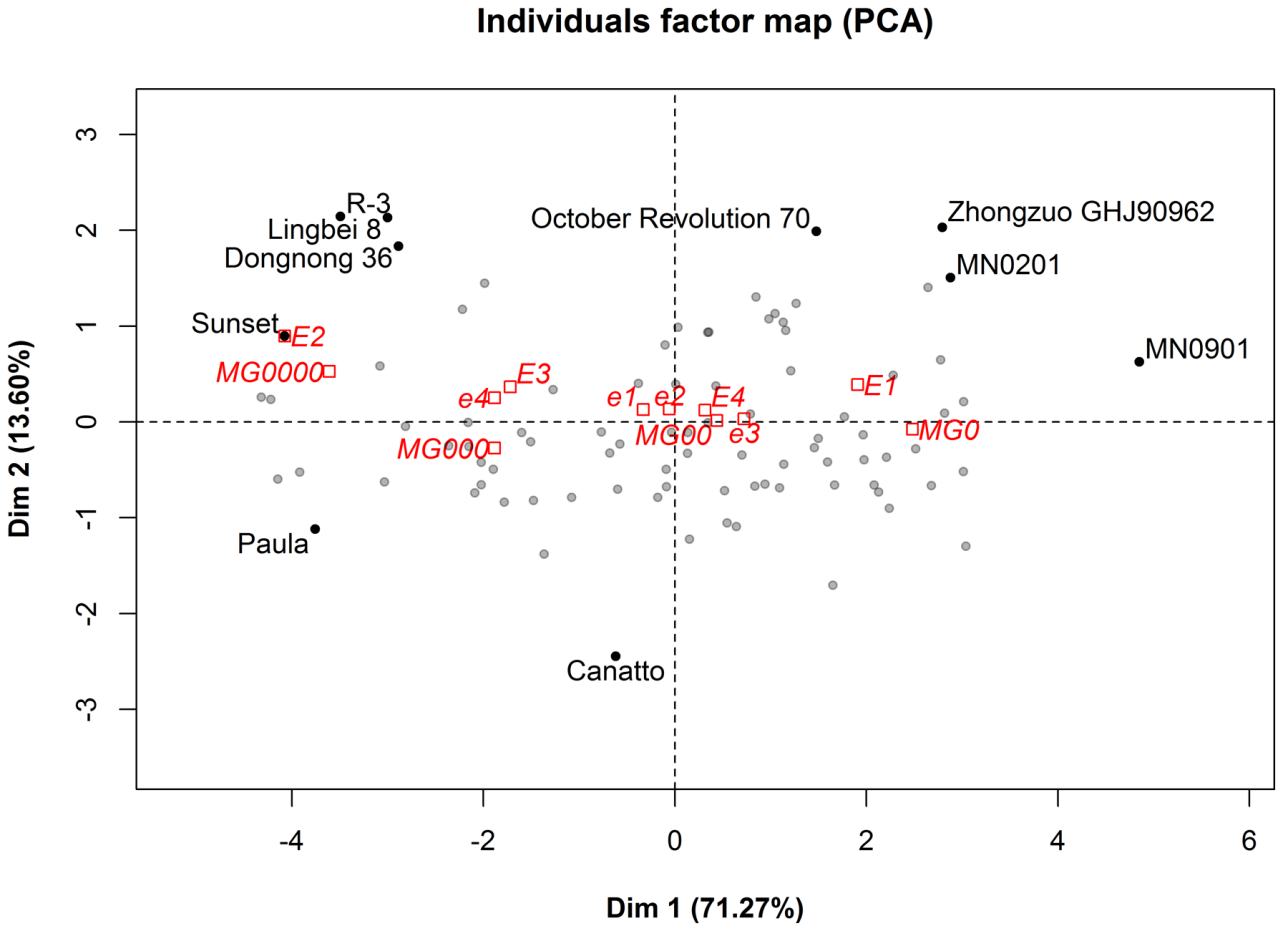
PCA individuals' factor map of soybeans from high-latitude cold regions. E1/e1, E2/e2, E3/e3, and E4/e4 are the four maturity loci marked in red. MG0, MG00, MG000 and MG0000 are the four maturity groups marked in red. The top ten contributor soybeans are marked in black.

PCA showed that the first two principal components of Dim 1 and 2 could explain 71.27% and 13.60% of the variation, respectively, and 84.87% in total (Figures 4 and 5). During the two years, VE-R7, VE-R1, and R1-R7 all showed positive correlations with the first principal component, Dim 1, whereas VE-R7 had the highest correlation (Figure 4). As for the second component, Dim 2, VE-R1 positively correlated with it while R1–R7 negatively correlated (Figure 4). That is to say, Dim 1 reflects maturity while Dim 2 relates with flowering. In Figure 5, the maturity groups MG0000, MG000, MG00 and MG0 dispersed along Dim 1. On Dim 1, the maturity loci *E1* and *E4* had larger coordinates than their recessive versions, *e1* and *e4*, whereas *E2* and *E4* were smaller than their recessive versions, *e2* and *e4*.

## Discussion

Until recently, HCRs were not viewed as suitable for soybean production because they exhibit low average temperatures and short frost-free periods that are not long enough for late-maturing varieties. However, with the variety improvement and development of farming technology, HCRs have become more and more important for the soybean industry under the background of global warming, the increasing population, and arable land shortages.

The soybean is a short-day crop and diversifies, in terms of maturity, among varieties. For convenience, soybean varieties are classified into different MGs to direct breeding and planting practices. However, although HCR soybeans are known to be relatively insensitive to photoperiods compared with those from other regions, information is still missing regarding their MGs. This information is missing partially because HCR soybeans mature much earlier and have a low differentiation degree at medium and low latitudes, where the main production areas currently are. However, as we know, few reports about MG have been conducted in HCRs.

In Heihe, the tested varieties displayed a broad diversity in their maturity (Figure 1, and Tables 2 and S1). MG0 matured partially, suggesting that HCR soybeans belong to MG0 or even more early-maturing MGs. Some HCR soybeans matured much earlier than MG000 and were clustered into the new MG0000. Therefore, HCR soybeans show diverse maturity rates.

HCR soybeans are relatively insensitive to the photoperiod. Thus, whether they can flower is not the main problem for maturation. In Table 4, the correlation between VE-R1 and VE-R7 or R1-R7 was less than that between VE-R7 and R1-R7, suggesting that the reproductive growth duration is much more important than the vegetative growth/duration for maturation. The PCA results also provide further evidence. The first principal component, Dim 1, related most significantly with maturity, whereas the second component, Dim 2, exhibited flowering time (Figure 4). The tested varieties predominantly dispersed along Dim 1, whereas they narrowed along Dim 2 (Figure 5), suggesting that the post-flowering period is much more important than the pre-flowering period in maturity diversification and classification and that post-flowering photoperiodic responses play an important role for HCR soybean maturation [40].

Soybean growth period traits not only include the maturity time but also its structure [1], i.e., the time to first-flowering, first-podding, etc., should also be considered. Egli found that seed yield is related to the length of the reproductive phase rather than the total growth duration [2]. For early maturing varieties, SD treatment before flowering significantly promotes post-flowering development but does not significantly promote pre-flowering development [41]. Our experiment also yielded similar results. In our experiment, Paula and Ha 6223-4 had the top two shortest VE-R1 in 2010 (Table S1), but belonged to different MGs (MG0000 and MG000 in Table 1, respectively), and MG0000 had the narrowest range of DFF (Figure 1b). These observations suggest that HCR soybeans have relatively stable DFF. Therefore, for HCR soybeans, which are typically early maturing, their vegetative growth periods are similar, but their reproductive periods are diverse (Table S1). This further suggests that lengthening the reproductive growth period would not hamper the vegetative growth nor inhibit dry matter accumulation [1] and that HCR soybean breeding should guarantee sufficient vegetative growth. Under high latitudes with low average temperatures and short frost-free periods, soybean breeding should be more focused on shortening post-flowering reproductive growth when vegetative growth is shortened to an extent that necessary vegetative accumulation can be guaranteed. At low latitudes or in tropical and subtropical settings, a short photoperiod is too promotional for soybean flowering and maturation, and a long juvenile trait is needed to lengthen the vegetative growth period to ensure enough vegetative accumulation to increase production [42], [43]. Therefore, in breeding, it is necessary to regulate vegetative and reproductive growth to adapt to different environments.

Some varieties matured much earlier than the MGR soybeans of MG000, which is known to be the earliest maturing MG (Table 3 and Figures 2 and 3). The distance between these early varieties and the MGR soybeans of MG000 is sufficient to determine a new MG, according to the conventions of MG classification. Star 4/75, Sunset, Dongnong 36, and Dongnong 41-C should be the MGR soybeans of this new MG because they have a relatively low SD/Mean value (Table S1).

Soybean maturity traits are under the control of multiple genes. Currently, nine related loci have been found [5]–[13], and their *E1*, *E2*, *E3* and *E4* have been molecularly identified [16]–[19]. Their reproductive periods were demonstrated to be controlled by one major gene, plus polygenes, in a research study involving nine crosses, and two major effector QTLs, *qRP-c-1* and *qRP-l-1*, (which associate with *E8* and *E3*, respectively) may function there [44]. In the 48 tested varieties, loci *E1* and *E2* were found to exist predominantly as their recessive alleles, and their dominant alleles, *E1* and *E2*, only distributed in 4 and 1 varieties, respectively (Table 5), suggesting that loci *E1* and *E2* have strong impacts on the adaption to HCRs. As for loci *E3* and *E4*, they appear to have different roles on maturity. In Figure 5, allele *E3* has a lower coordinate on Dim 1 than its recessive allele, *e3*, whereas allele *E4* has a higher coordinate than its recessive allele, *e4*. Thus, allele *E3* promotes maturation while allele *E4* delays it. From their coordinates on Dim 2, it appears that *E4*/*e4* does not have a role in flowering, whereas *E3* delays and *e3* promotes flowering. The converse roles of two phytochrome genes, *E3* and *E4*, might be related with the low average temperatures in HCRs. In addition, each MG had multiple genotypes, and one genotype could distribute into several MGs. Thus, the number of recessive alleles did not correlate with the MG classification in the HCRs. These four MGs harbor the diversity of genotypes of E genes, indicating that, in HCRs, the mechanism of maturity is rather complex and involves more genes than these four E genes.

## Supporting Information

File S1 Raw data of soybean maturity traits in 2010 and 2011.
(XLS)

Table S1 Growth periods of the tested soybean varieties.
(DOC)
