# Supplementary material for: Maturity Group Classification and Maturity Locus Genotyping of Early-Maturing Soybean Varieties from High-Latitude Cold Regions
Source: PLoS One. 2014 Apr 16;9(4):e94139. doi: 10.1371/journal.pone.0094139 (PMC3989213; doi:10.1371/journal.pone.0094139)
Supplement: Table S1 — Growth periods of the tested soybean varieties. (DOC) [file pone.0094139.s002.doc]

**Table S1. Growth periods of the tested soybean varieties**

|  | **2010** | | | **2011** | | |  |
| --- | --- | --- | --- | --- | --- | --- | --- |
| **Soybean Variety** | **VE-R7** | **VE-R1** | **R1-R7** | **VE-R7** | **VE-R1** | **R1-R7** | **Variation/Mean** |
| Amur 262 | 104.1±0.4 | 31.8±0.5 | 72.3±0.6 | 98.1±0.6 | 40.1±0.6 | 58.0±0.5 | 0.06 |
| Amur 283 | 103.6±0.6 | 32.9±0.7 | 70.7±0.7 | 100.4±1.0 | 39.6±0.7 | 60.8±0.6 | 0.03 |
| Bei 02-7495 | 101.3±0.7 | 32.6±1.0 | 68.7±0.7 | 92.0±1.0 | 35.3±1.4 | 56.7±1.4 | 0.10 |
| Bei 1249 | 116.4±0.8 | 30.8±1.2 | 85.6±0.8 | 108.0±1.0 | 38.1±1.3 | 69.9±1.2 | 0.07 |
| Beidou 16 | 101.0±1.1 | 30.7±1.1 | 70.3±0.8 | 92.3±1.2 | 34.6±1.5 | 57.7±1.5 | 0.09 |
| Beidou 19 | 111.5±0.8 | 30.7±1.0 | 80.8±1.0 | 104.7±1.0 | 37.6±1.5 | 67.1±1.0 | 0.06 |
| Beidou 24 | 98.2±1.1 | 29.7±1.0 | 68.5±0.5 | 98.9±0.8 | 36.6±1.5 | 62.3±1.6 | 0.01 |
| Beidou 37 | 110.0±0.5 | 38.5±0.6 | 71.5±0.6 | 110.1±0.8 | 39.0±0.9 | 71.1±0.8 | 0.00 |
| Beidou 8 | 106.4±0.9 | 33.0±0.9 | 73.4±0.8 | 104.7±0.5 | 37.6±1.1 | 67.1±1.1 | 0.02 |
| Beifeng 1 | 98.2±0.6 | 28.0±0.5 | 70.2±0.4 | 90.5±0.7 | 34.1±1.1 | 56.3±0.9 | 0.08 |
| Bista | 90.1±0.6 | 26.5±1.0 | 63.6±0.8 | 82.6±0.9 | 34.0±1.4 | 48.6±1.5 | 0.09 |
| Canatto | 97.8±0.9 | 35.2±0.7 | 62.6±0.7 | 92.0±0.8 | 27.0±0.8 | 65.0±0.0 | 0.06 |
| Dengke 2 | 87.6±0.9 | 27.9±1.2 | 59.7±0.9 | 82.7±0.8 | 37.7±0.7 | 45.0±0.9 | 0.06 |
| Dewdrop | 96.7±0.7 | 29.0±0.6 | 67.7±0.6 | 95.1±0.5 | 38.7±0.7 | 56.4±0.7 | 0.02 |
| Dongnong 36 | 85.5±0.9 | 30.6±1.1 | 54.8±0.9 | 77.3±0.7 | 38.2±0.8 | 39.1±1.0 | 0.10 |
| Dongnong 40 | 97.8±0.9 | 30.7±0.8 | 67.1±0.4 | 98.2±0.8 | 39.1±1.2 | 59.0±1.4 | 0.00 |
| Dongnong 41 | 84.3±1.1 | 30.1±1.0 | 54.2±0.5 | 77.9±0.6 | 34.4±0.6 | 43.5±0.6 | 0.08 |
| Dongnong 41-C | 84.0±0.8 | 25.9±0.8 | 58.1±0.6 | 81.8±0.7 | 33.9±1.0 | 47.9±1.0 | 0.03 |
| Fengshou 15 | 111.4±1.2 | 31.6±1.2 | 79.7±0.7 | 104.3±1.0 | 36.2±1.3 | 68.2±1.0 | 0.07 |
| Fengshou 23 | 102.2±0.8 | 31.5±1.0 | 70.7±1.1 | 91.3±0.7 | 33.9±0.9 | 57.4±0.8 | 0.11 |
| Fengshou 24 | 111.2±1.7 | 31.3±1.7 | 79.9±0.7 | 98.9±1.1 | 36.7±1.3 | 62.2±1.2 | 0.12 |
| Fengshou 26 | 110.8±1.2 | 30.4±1.3 | 80.4±1.0 | 106.3±0.9 | 36.1±1.3 | 70.1±1.0 | 0.04 |
| Fengshou 27 | 111.0±1.4 | 40.9±1.7 | 70.1±0.9 | 109.9±0.7 | 36.8±0.8 | 73.2±0.9 | 0.01 |
| Glacier | 107.3±1.1 | 35.3±0.9 | 72.0±0.7 | 95.2±0.8 | 34.0±1.1 | 61.2±1.1 | 0.12 |
| Gritiaz 80 | 103.9±1.0 | 34.5±0.6 | 69.4±1.0 | 98.7±0.9 | 39.6±1.1 | 59.1±1.3 | 0.05 |
| Ha 6223-4 | 92.9±0.6 | 24.8±0.7 | 68.0±0.8 | 84.3±1.0 | 33.2±1.4 | 51.1±1.1 | 0.10 |
| Harmony | 104.5±0.7 | 32.8±0.6 | 71.7±0.7 | 100.3±0.7 | 40.6±0.7 | 59.7±0.6 | 0.04 |
| Hefeng 37 | 98.8±1.2 | 31.3±1.1 | 67.5±1.1 | 93.8±0.7 | 33.4±1.2 | 60.5±1.3 | 0.05 |
| Heihe 11 | 98.7±0.8 | 32.2±0.7 | 66.6±0.9 | 85.0±1.3 | 32.3±1.2 | 52.6±0.8 | 0.15 |
| Heihe 12 | 94.6±0.9 | 31.7±0.7 | 62.9±0.8 | 85.9±1.5 | 35.4±1.0 | 50.5±1.7 | 0.10 |
| Heihe 13 | 99.9±0.8 | 28.9±0.7 | 71.0±0.7 | 97.7±0.5 | 38.1±0.7 | 59.6±0.7 | 0.02 |
| Heihe 14 | 90.0±0.7 | 25.5±0.6 | 64.5±0.6 | 82.4±1.1 | 35.0±1.7 | 47.4±1.9 | 0.09 |
| Heihe 18 | 112.6±0.7 | 31.5±0.8 | 81.1±0.8 | 100.9±1.0 | 37.9±0.5 | 63.0±1.0 | 0.11 |
| Heihe 20 | 93.0±1.2 | 29.9±1.1 | 63.1±1.0 | 86.8±1.1 | 32.2±1.3 | 54.6±0.7 | 0.07 |
| Heihe 28 | 93.4±0.6 | 28.4±0.7 | 65.0±0.5 | 85.5±0.8 | 34.4±1.3 | 51.2±1.5 | 0.09 |
| Heihe 3 | 102.4±1.2 | 31.1±0.8 | 71.4±0.9 | 97.8±0.9 | 33.5±1.9 | 64.2±1.7 | 0.05 |
| Heihe 33 | 97.9±1.1 | 26.6±1.3 | 71.3±0.8 | 89.6±1.1 | 35.5±1.4 | 54.1±1.3 | 0.09 |
| Heihe 35 | 91.6±1.2 | 29.2±1.4 | 62.4±0.9 | 82.6±1.0 | 32.3±1.2 | 50.4±1.0 | 0.10 |
| Heihe 36 | 115.5±1.0 | 31.5±1.9 | 84.0±2.0 | 109.9±0.9 | 35.6±1.6 | 74.4±1.1 | 0.05 |
| Heihe 37 | 103.2±0.7 | 27.2±0.7 | 76.0±0.7 | 93.6±0.7 | 33.7±1.4 | 59.9±1.2 | 0.10 |
| Heihe 38 | 115.3±1.3 | 31.2±1.0 | 84.1±1.1 | 105.9±0.8 | 37.2±1.5 | 68.7±1.2 | 0.08 |
| Heihe 39 | 105.3±1.3 | 28.1±1.5 | 77.1±1.4 | 96.2±0.7 | 34.4±1.4 | 61.9±1.3 | 0.09 |
| Heihe 41 | 91.6±1.2 | 29.6±1.1 | 62.0±1.0 | 86.2±0.7 | 34.2±1.7 | 52.0±1.7 | 0.06 |
| Heihe 43 | 111.3±1.1 | 30.4±1.1 | 80.9±0.8 | 99.8±0.8 | 36.3±1.6 | 63.6±1.3 | 0.11 |
| Heihe 44 | 91.4±0.8 | 28.4±0.9 | 63.1±0.6 | 82.6±0.6 | 33.2±1.7 | 49.4±1.5 | 0.10 |
| Heihe 45 | 108.7±0.8 | 31.0±1.1 | 77.7±0.8 | 96.7±0.5 | 35.4±1.1 | 61.3±1.2 | 0.12 |
| Heihe 46 | 109.3±1.1 | 31.1±0.9 | 78.2±1.0 | 104.5±0.8 | 37.1±1.0 | 67.4±0.6 | 0.04 |
| Heihe 48 | 112.5±1.1 | 31.4±1.1 | 81.0±0.8 | 106.7±1.2 | 38.0±1.3 | 68.6±1.0 | 0.05 |
| Heihe 49 | 88.5±0.9 | 26.9±0.9 | 61.6±0.9 | 75.8±0.8 | 31.9±1.2 | 43.9±0.9 | 0.16 |
| Heihe 5 | 107.2±1.3 | 29.9±1.3 | 77.3±0.9 | 98.4±0.6 | 36.4±0.9 | 62.0±0.8 | 0.09 |
| Heihe 50 | 106.3±1.2 | 27.4±0.7 | 78.9±0.9 | 97.8±0.7 | 41.4±1.5 | 56.4±1.1 | 0.08 |
| Heihe 51 | 105.7±0.9 | 31.5±0.9 | 74.1±0.8 | 98.1±0.7 | 35.1±1.0 | 63.0±1.1 | 0.07 |
| Heihe 7 | 98.3±1.0 | 29.9±1.2 | 68.4±0.6 | 97.0±0.8 | 35.4±1.3 | 61.6±1.1 | 0.01 |
| Heihe 8 | 97.3±1.1 | 30.6±0.8 | 66.8±0.8 | 90.8±0.6 | 34.8±1.1 | 56.0±1.1 | 0.07 |
| Heihe 9 | 103.7±0.8 | 33.4±0.6 | 70.3±0.7 | 96.8±0.9 | 40.2±0.8 | 56.6±0.8 | 0.07 |
| Huajiang 2 | 103.4±0.8 | 39.8±1.3 | 63.6±1.0 | 95.6±0.6 | 32.2±1.0 | 63.4±0.9 | 0.08 |
| Huajiang 3 | 121.2±1.0 | 49.8±1.1 | 71.3±1.1 | 101.3±0.7 | 35.4±1.2 | 65.9±1.4 | 0.18 |
| Hujiao 07-2123 | 79.1±1.2 | 25.5±1.2 | 53.6±0.6 | 74.0±1.4 | 30.9±0.7 | 43.1±1.4 | 0.07 |
| Hujiao 07-2479 | 78.9±1.0 | 26.0±0.7 | 53.0±1.0 | 76.3±0.5 | 31.2±0.8 | 45.1±0.6 | 0.03 |
| Jiangmodou 1 | 112.1±0.5 | 40.9±0.6 | 71.1±0.7 | 103.2±1.0 | 37.5±1.2 | 65.7±0.8 | 0.08 |
| Jilin 30 | NA | 49.3±0.9 | NA | NA | 54.6±0.5 | NA | NA |
| Jiufeng 10 | 106.6±0.9 | 30.2±1.5 | 76.4±1.2 | 102.0±1.0 | 37.1±1.5 | 64.8±1.2 | 0.04 |
| Jiufeng 7 | 107.5±1.0 | 26.7±1.0 | 80.8±0.6 | 101.6±0.6 | 38.4±1.3 | 63.2±1.2 | 0.06 |
| Jiufeng 9 | 111.5±1.1 | 30.4±1.6 | 81.0±1.3 | 103.8±0.8 | 36.7±1.0 | 67.1±0.9 | 0.07 |
| Jiunong 21 | NA | 47.3±0.9 | NA | NA | 51.7±0.5 | NA | NA |
| Kennong 8 | 91.6±0.6 | 31.4±0.7 | 60.2±0.6 | 83.6±0.9 | 32.3±1.8 | 51.2±1.8 | 0.09 |
| Lingbei 8 | 82.5±0.7 | 25.9±0.7 | 56.6±0.7 | 79.1±0.7 | 40.1±0.9 | 38.9±0.9 | 0.04 |
| Lydia | 98.1±0.5 | 36.1±0.8 | 62.0±0.8 | 97.6±0.6 | 37.7±0.7 | 59.9±0.7 | 0.01 |
| Maple Presto | 88.5±0.9 | 27.1±0.7 | 61.4±0.5 | 82.0±1.4 | 33.6±1.3 | 48.4±1.6 | 0.08 |
| Maple Ridge | 95.9±0.8 | 38.9±1.1 | 57.0±1.1 | 96.3±0.6 | 36.7±0.7 | 59.6±0.6 | 0.00 |
| Mengdou 11 | 103.6±0.9 | 31.1±1.1 | 72.5±0.8 | 97.4±0.6 | 35.8±0.8 | 61.6±0.6 | 0.06 |
| Mengdou 30 | 109.5±0.6 | 35.1±0.6 | 74.4±0.9 | 108.7±0.7 | 42.0±0.7 | 66.7±0.7 | 0.01 |
| Mengdou 31 | 95.6±0.6 | 30.3±0.6 | 65.3±0.7 | 97.6±0.7 | 37.2±0.8 | 60.4±0.8 | 0.02 |
| Mengdou 7 | 97.8±1.1 | 28.7±1.0 | 69.1±0.7 | 88.5±0.9 | 35.5±1.6 | 52.9±1.3 | 0.10 |
| Mengdou 9 | 106.1±0.9 | 30.2±0.6 | 76.0±0.8 | 95.4±0.6 | 37.5±0.8 | 57.9±1.0 | 0.11 |
| MN0201 | 120.4±1.0 | 47.8±1.8 | 72.6±2.0 | 99.4±0.7 | 39.6±1.3 | 59.8±1.3 | 0.19 |
| MN0901 | 124.9±1.2 | 48.8±2.4 | 76.1±2.3 | 115.4±0.6 | 38.8±1.3 | 76.6±1.3 | 0.08 |
| OAC Vision | 93.8±0.4 | 28.7±0.5 | 65.1±0.3 | 87.8±0.9 | 31.0±0.8 | 56.8±0.6 | 0.07 |
| October Revolution 70 | 104.8±0.8 | 35.2±0.7 | 69.6±0.9 | 101.1±0.7 | 42.4±0.7 | 58.7±0.7 | 0.04 |
| Paula | 79.8±0.7 | 24.6±0.7 | 55.2±0.6 | 77.8±1.6 | 30.0±0.7 | 47.8±1.5 | 0.03 |
| R-1 | 105.4±0.7 | 33.2±0.7 | 72.2±0.8 | 99.6±0.6 | 38.4±0.5 | 61.2±0.6 | 0.06 |
| R-2 | 89.7±0.5 | 25.8±0.7 | 63.8±0.5 | 84.2±0.8 | 32.8±1.2 | 51.4±1.4 | 0.06 |
| R-3 | 80.0±0.5 | 26.5±0.6 | 53.5±0.6 | 76.6±0.9 | 39.4±1.4 | 37.1±1.6 | 0.04 |
| R-4 | 78.3±0.5 | 26.0±1.0 | 52.3±0.9 | 72.1±0.9 | 33.1±1.5 | 39.0±1.4 | 0.08 |
| Sonata | 89.7±0.6 | 28.7±0.6 | 61.0±0.5 | 82.6±0.8 | 38.6±0.9 | 44.0±1.1 | 0.08 |
| Star 4/75 | 78.2±0.5 | 25.7±0.7 | 52.4±0.6 | 73.3±1.3 | 33.2±1.0 | 40.1±1.5 | 0.06 |
| Suinong 14 | NA | 39.4±1.1 | NA | NA | 42.0±0.6 | NA | NA |
| Sunset | 77.9±0.8 | 26.4±0.9 | 51.5±1.1 | 74.2±0.9 | 35.1±0.7 | 39.1±0.7 | 0.05 |
| Sunset 1 | 97.1±0.8 | 33.0±0.6 | 64.1±0.8 | 92.1±0.6 | 36.2±0.7 | 55.9±0.9 | 0.05 |
| Surge | NA | 42.8±0.7 | NA | NA | 42.2±0.8 | NA | NA |
| Terek | 103.4±0.6 | 30.5±0.6 | 72.9±0.6 | 91.8±0.6 | 36.1±0.4 | 55.7±0.7 | 0.12 |
| Traill | 113.1±1.2 | 29.9±1.0 | 83.2±1.0 | 99.1±0.7 | 33.4±1.3 | 65.7±1.3 | 0.13 |
| Zhongzuo GHJ90962 | 108.9±0.4 | 34.6±0.5 | 74.3±0.4 | 110.1±0.8 | 44.1±0.7 | 66.0±0.7 | 0.01 |
